# *Plasmodium falciparum* chloroquine resistance transporter (PfCRT) isoforms PH1 and PH2 perturb vacuolar physiology

**DOI:** 10.1186/s12936-016-1238-1

**Published:** 2016-03-31

**Authors:** Paul S. Callaghan, Amila Siriwardana, Matthew R. Hassett, Paul D. Roepe

**Affiliations:** Department of Chemistry, Georgetown University, 37th and O Streets, NW, Washington, DC 20057 USA; Department of Biochemistry, Cellular and Molecular Biology, Georgetown University, 37th and O Streets, NW, Washington, DC 20057 USA

**Keywords:** Chloroquine resistance, Drug transport, Cytostatic

## Abstract

**Background:**

Recent work has perfected yeast-based methods for measuring drug transport by the *Plasmodium falciparum* chloroquine (CQ) resistance transporter (PfCRT).

**Methods:**

The approach relies on inducible heterologous expression of PfCRT in *Saccharomyces cerevisiae* yeast. In these experiments selecting drug concentrations are not toxic to the yeast, nor is expression of PfCRT alone toxic. Only when PfCRT is expressed in the presence of CQ is the growth of yeast impaired, due to inward transport of chloroquine (CQ) via the transporter.

**Results:**

During analysis of all 53 known naturally occurring PfCRT isoforms, two isoforms (PH1 and PH2 PfCRT) were found to be intrinsically toxic to yeast, even in the absence of CQ. Additional analysis of six very recently identified PfCRT isoforms from Malaysia also showed some toxicity. In this paper the nature of this yeast toxicity is examined. Data also show that PH1 and PH2 isoforms of PfCRT transport CQ with an efficiency intermediate to that catalyzed by previously studied CQR conferring isoforms. Mutation of PfCRT at position 160 is found to perturb vacuolar physiology, suggesting a fitness cost to position 160 amino acid substitutions.

**Conclusion:**

These data further define the wide range of activities that exist for PfCRT isoforms found in *P. falciparum* isolates from around the globe.

**Graphical abstract:**
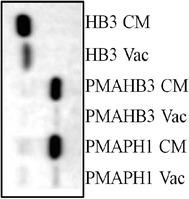
V5 Western blot quantifying relative PfCRT expression in total (CM) vs vacuolar (VAC) yeast membranes for unmodified HB3 PfCRT vs HB3 and PH1 PMA–PfCRT chimeras.

## Background

Since the discovery of *Plasmodium falciparum* chloroquine (CQ) resistance transporter PfCRT and its role as the primary genetic determinant of chloroquine resistance (CQR) in *P. falciparum*, 53 distinct isoforms of this 424 amino acid protein have been found to be expressed in parasite isolates from around the globe [1–4]. These have descended from at least five independent ‘founder events’ in four regions: Southeast Asia (SEA), South America (SA) (two events), Papua New Guinea (PNG), and the Philippines [5, 6], presumably via variable drug selection pressure in these regions. Patterns of amino acid substitutions and the degree of drug resistance conferred by the isoforms segregate in part based on their geographic origin. For example, isoforms from SEA typically harbour six to eight substitutions and confer high-level CQR to SEA *P. falciparum* isolates, whereas SA isoforms tend to harbour four to five substitutions and confer more moderate levels of CQR [7, 8]. However, even within one geographical region resistance of parasite isolates to different drugs, as well as other characteristics, can vary considerably, yet the function of many PfCRT isoforms expressed in these isolates has not yet been studied. Thus, although PfCRT is clearly essential for CQR, it is unclear what precise level of CQR, as well as what form(s) of multidrug resistance, can be conferred by many PfCRT isoforms [3, 4].

Even after the widespread withdrawal of CQ as an approved treatment against *P. falciparum* malaria, mutant CQR-conferring PfCRT isoforms have persisted in some regions (especially within SA) [8]. This has been attributed to several factors including: PfCRT’s putative role in cross-resistance to other quinoline drugs that are still in use, the continued use of CQ to treat *P. vivax* malaria in these same regions, acquisition of compensatory genetic alterations that confer unique fitness phenotypes that require the mutant PfCRT, and most recently, the apparent importance of certain PfCRT mutations for creating backgrounds in which delayed artemisinin clearance phenotypes can develop more readily [9]. A fifth possibility suggested by recent work is that some mutant PfCRT isoforms are revertants that do not confer CQR at all [4, 10–12], which if validated would have significant impact on current drug use policies and might also add to the interpretation of data from a variety of earlier field based studies (e.g. [13]).

In 2003, Chen and colleagues reported on a putative CQR founder event in the Philippines [14]. This study reported the sequences of PfCRT isoforms ‘PH1’ and ‘PH2’, which harbour novel mutations A144T and L160Y. Subsequent microsatellite analysis proved that these isoforms were in fact new examples of PfCRT evolved to harbour the key mutation K76T within a different parasite genetic background [6]. Presence of the K76T mutation within PfCRT has until very recently been considered ‘diagnostic’ for a CQR phenotype, so it is assumed that PH1 and PH2 confer CQR [4, 6]. However, direct analysis of the function of these isoforms is required to test this conclusion, particularly since possible exceptions to CQR status for parasites expressing K76T mutant PfCRTs have been noted [10–12].

Globally, PH1 and PH2 PfCRT isoforms are quite scarce and to our knowledge have only been found in the Philippines. However, for reasons that are not entirely clear, they are relatively abundant there as shown in a recent screen of 70 Filipino isolates wherein 33 harboured PH1 PfCRT (47 %) and 15 harboured PH2 (21 %) [6]. Single-pass drug susceptibility assays on parasite isolates expressing PH1 PfCRT indicated low-level CQR [13]. However, drug susceptibility measurements with parasite isolates (as opposed to more well behaved strains established in laboratory culture) often show wider variability in computed drug IC_50_ values. Isolates expressing PH2 could not be established in culture long enough for any drug susceptibility data to be obtained [14]. Thus, it remains unclear precisely what drug resistance phenotypes could be conferred by expression of these PfCRT isoforms in different *P. falciparum* genetic backgrounds.

One possible explanation for why PH1 and PH2 have not been seen in other geographic regions is the presence of an unusual L160Y mutation in these PfCRTs. Most amino acid substitutions associated with other PfCRT isoforms require only a single base change from the wild type coding sequence [e.g., for SA isoforms typically: C(tgt)72 → S(tct or agt), K(aaa)76 → T(aca), A(gcc)144 → T(tcc), A(gcc)220 → S(tcc), N(aac)326 → D(gac), I(ata)356 → L(tta)]. However, mutation L160Y in PH1 and PH2 requires two base changes [L(ctt)160 → Y(tat)]. This is true even if the parasite first acquires silent mutations to create any of the other five leucine codons. The only other two base mutations associated with amino acid substitutions in PfCRT isoforms are those that confer mutations A144F (found in Cam 734 and IsoV PfCRT), A144Y (found in China B, C, D PfCRT) and the N75E mutation found in Dd2 PfCRT and others from SEA [4]. The A144 mutation is quite rare [4] and it is thought that the more common N75E substitution may have arisen via two single base change steps (first to create homologous N75D, and then D75E, each of which requires only one mutation).

As mentioned, until recently the CQR vs CQS status of parasite isolates has often been assigned based on the presence of the key PfCRT K76T mutation alone [4]. In many cases however, these assignments are made without companion in vitro drug susceptibility measurements. This practice may have been sufficient when CQ was widely used and CQ selective pressure was high, but K76T may no longer be a fully reliable predictor of CQR [4, 10–12]. For example, in one recent field study 96 % of the isolates sequenced were 76T, but only 20 % of these demonstrated even moderate resistance to CQ [12]. Clearly, it is crucial to monitor the continued evolution of PfCRT protein both to inform an understanding of its interaction with currently used anti-malarials and to assess potential re-introduction of CQ as a regional treatment (perhaps as a companion drug in geographically constrained combination therapy). In this paper an initial investigation of the function of PfCRT isoforms PH1 and PH2, as well as others that show similar mutations relative to PH1 and PH2, is presented.

## Methods

### Materials

Yeast DOB were obtained in powder form from MP Biomedicals (Solon, OH, USA). Cell culture plastics were from BD Falcon (San Jose, CA, USA). Dextrose, galactose, and raffinose were obtained from Sigma (St. Louis MO, USA). Glass beads for yeast cell lysis were from B. Braun Biotech (Allentown, PA, USA). Anti-V5-HRP antibody was from Invitrogen (Carlsbad, CA, USA). Mutagenesis reagents were obtained from Agilent (Santa Clara, CA, USA). All other chemicals were reagent grade or better, were purchased from Sigma (St. Louis MO, USA) and used without additional purification.

### Yeast strains

CH1305 (MAT**a***ade2 ade3 ura3*-*52 leu2 lys2*-*801*) was provided by Cannon [15]. ΔVma (MATa *leu2Δ0 met15Δ0 ura3Δ0)* and parental strain *Saccharomyces cerevisiae* BY4741 (MATa *his3Δ1 leu2Δ0 met15Δ0 ura3Δ0)* were from the non-essential yeast knockout MATa haploid collection which is commercially available through Fisher Scientific. Solid and liquid media were prepared as described in Sherman et al. [16], and included synthetic complete (SC) media lacking one or more specified amino acids, as well as rich medium (YPD). Induction of PfCRT protein expression, standard yeast growth methods, yeast transfections, and other routine methods were as described [3, 4, 17].

### Plasmids

The pYES2 backbone containing PfHB3vh, PfDd2vh, Pf7G8vh, and PMA-PfHB3vh was constructed previously [17] and these were used as template DNA in subsequent rounds of multisite-directed mutagenesis via the Agilent QUICK Change method to create the various isoforms of PfCRT (see Table 1). All constructs were confirmed by direct DNA sequencing of the full ‘yeast-optimized’ *pfcrt* gene.

**Table 1:** Amino acid sequences of PfCRT strains and isolates referenced in this study

Residues mutated relative to wild type are highlighted green. Where multiple CQ IC50 values (nM) were found in the literature, the high and low values are reported (Low, High)

### Western blotting

Western blotting was performed as described previously [3].

### Colony formation assays and quantitative growth analysis

**Fig. 1:**
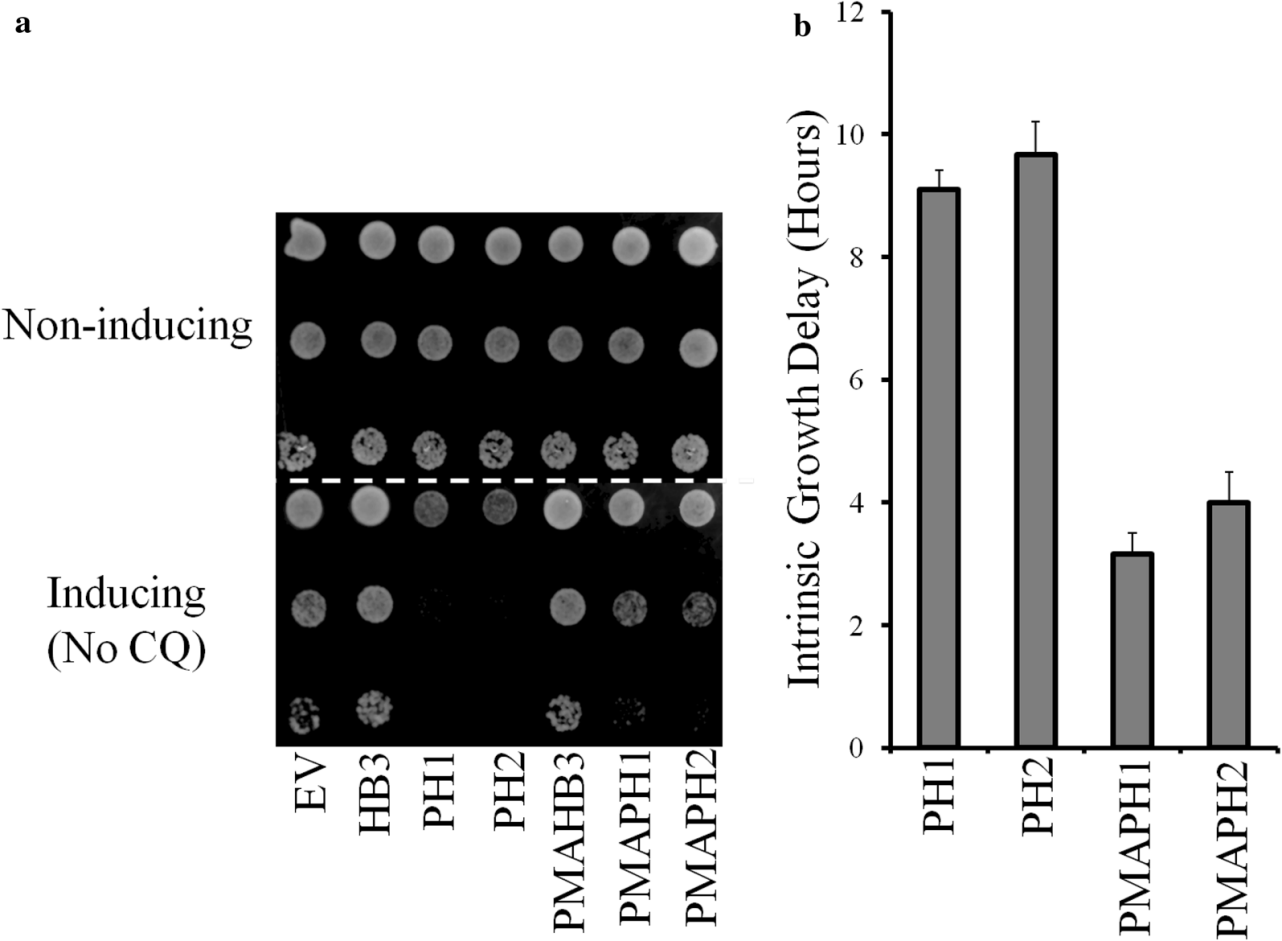
PH1 and PH2 expression is intrinsically toxic to CH1305 yeast. **a** Colony formation assay in which cells were plated on non-inducing media (SD/-URA, top) or inducing media lacking CQ (SGR/-URA, bottom). Expression of PH1 and PH2 PfCRT causes slowed growth in yeast in the absence of drug that is substantially mitigated by targeting increased expression to the PM/decreased expression at the vacuole. **b** Intrinsic growth delay calculated by taking the difference in time needed to reach maximal growth rate in inducing vs non-inducing media. Results reported relative to control yeast expressing HB3 PfCRT, Baro et al. [17]

Assays were performed under PfCRT-inducing and non-inducing conditions as described [3, 4, 17]. In brief, growth under each condition was measured for three independent clones in triplicate via back dilution of the strain grown under normal non-inducing conditions (SD media lacking uracil). Intrinsic growth delays were calculated by measuring the time to reach mid-exponential growth under PfCRT-inducing vs non-inducing conditions [17]. CQ-dependent growth delay conferred by PMAPH1 and PMAPH2 PfCRTs was calculated as described previously [17] with an additional correction subtracting the small intrinsic growth delay (Fig. 1) conferred by these isoforms.

### Yeast vacuole pH measurements

Yeast vacuole pH measurements were performed as described elsewhere [18–20]. In brief, cells were grown in inducing media (SGR/-URA) to mid-exponential phase. They were harvested, washed three times, and resuspended to an OD_600_ of 2.5 in APG media (10 mM arginine, 8 mM phosphoric acid, 2 % galactose, 2 mM MgSO_4_, 1 mM KCl, 0.2 mM CaCl_2_, pH 4) with 50 μM BCECF-AM. The solution was then incubated for 20 min at 30 °C, the cells were washed three times and resuspended in APG to an OD_600_ of 0.5, and fluorescence emission intensity was measured at 535 nm with excitation at 490 nm.

Calibration of probe response was done for each experiment involving fresh yeast cultures by suspending parent cells transformed with empty vector (EV) in calibration buffers [50 mM Mes, 50 mM HEPES, 50 mM KCl, 50 mM NaCl, 0.2 mM ammonium acetate, 10 mM sodium azide, 10 mM 2-deoxy glucose, 50 μM carbonyl cyanide m-chlorophenyl hydrazone (CCCP)] at pH set from to 4.00 to 7.00 at 1.00 unit intervals [19, 20]. pH was determined by referencing sample intensities to the calibration curve generated the same day under parallel conditions.

### Yeast vacuole isolation

Yeast vacuoles were isolated as described previously [21], with some modifications. In brief, yeast cultures were grown in SGR-ura to mid-exponential phase and cells were harvested at 3000 g for 5 min. The cell pellet was resuspended in 50 mL wash buffer [100 mM Tris–Cl (pH 9.4), 100 mM DTT], cells were incubated at 30 °C for 10 min and gently mixed at 2-min intervals. Cells were pelleted at 3000 g for 3 min and resuspended in 15 mL spheroplasting buffer (50 mM KPO_4_, 0.15 % YPD, 600 mM sorbitol) via gentle vortexing. Five-hundred microliter of zymolase solution [zymolase 20 T (300 mg/mL) in spheroplasting buffer] was added and gently swirled into the cell suspension, cells were incubated for 1 h at 30 °C with gentle swirling at 5-min intervals; the resulting spheroplasts were transferred to a chilled 50 mL conical tube and pelleted at 1000 g for 2 min. The spheroplast pellet was resuspended in 2.5 mL of 15 % ficoll prepared in a 20 mM Pipes (pH 6.8)/200 mM sorbitol solution; 1.5 mg dextran was then swirled into the suspension which was then incubated on ice for 5 min while swirling every 30 s followed by 2-min incubation at 30 °C. Five milliliter of the lysate/15 % ficoll solution was pipetted to the bottom of a high-speed (SW-41) centrifuge tube. A ficoll step gradient was pipetted over the sample (3.0 mL 8 % ficoll followed by 3.0 mL of 4 % ficoll to within about 1 cm of the top of the tube). The tube was filled to within 3 mm of the rim with 0 % ficoll solution. The tubes were centrifuged at 175,000 rpm for 90 min at 4 °C and yeast vacuoles were recovered from the interface of the 0 and 4 % layers of the gradient. Purified vacuoles were stored at −80 °C after adding 50 % glycerol stock (to a final concentration of 10 % glycerol) supplemented with protease inhibitor cocktail, and snap-frozen over dry ice/ethanol.

### Parasite cell culture

Parasites were grown in sealed flasks at 37 °C in a 5 % CO2/5 % O2/90 % N2 atmosphere at 2 % haematocrit in RPMI-1640 medium supplemented with 0.5 % AlbuMAX II (Gibco/Invitrogen) [22, 23].

### Parasite synchronization

Cultures used in this work were routinely synchronized every three cycles with a 5 % d-sorbitol treatment which lyses mid-stage and late trophozoites as well as schizonts but leaves uninfected red blood cells (RBC), rings, and some early trophozoites unscathed [22]. Multiple synchronization treatments successively improve the ring: early trophozoite ratio, thus synchronization was routinely done three times. For localizing dextran-conjugated Oregon green 488 (OGd) to the parasite digestive vacuole (DV), methods used previously were followed [23].

### Single-cell photometry

Single-cell photometry (SCP) methods for measuring parasite DV pH were as described in detail previously [22, 23] using custom microscopy systems comprised of a Nikon diaphot microscope, a Photometrics Sensys 12-bit CCD camera, associated optics, custom perfusion cells, and custom dynamic thresholding software.

### Spinning disk confocal microscopy

Data acquisition, deconvolution, 3D restoration, and parasite DV volume measurements were done as described in detail previously [22, 24] using a customized Perkin–Elmer spinning disk confocal microscope with a 491 nm laser line, typically at 200 ms exposure and 35 % laser power.

## Results

**Fig. 2:**
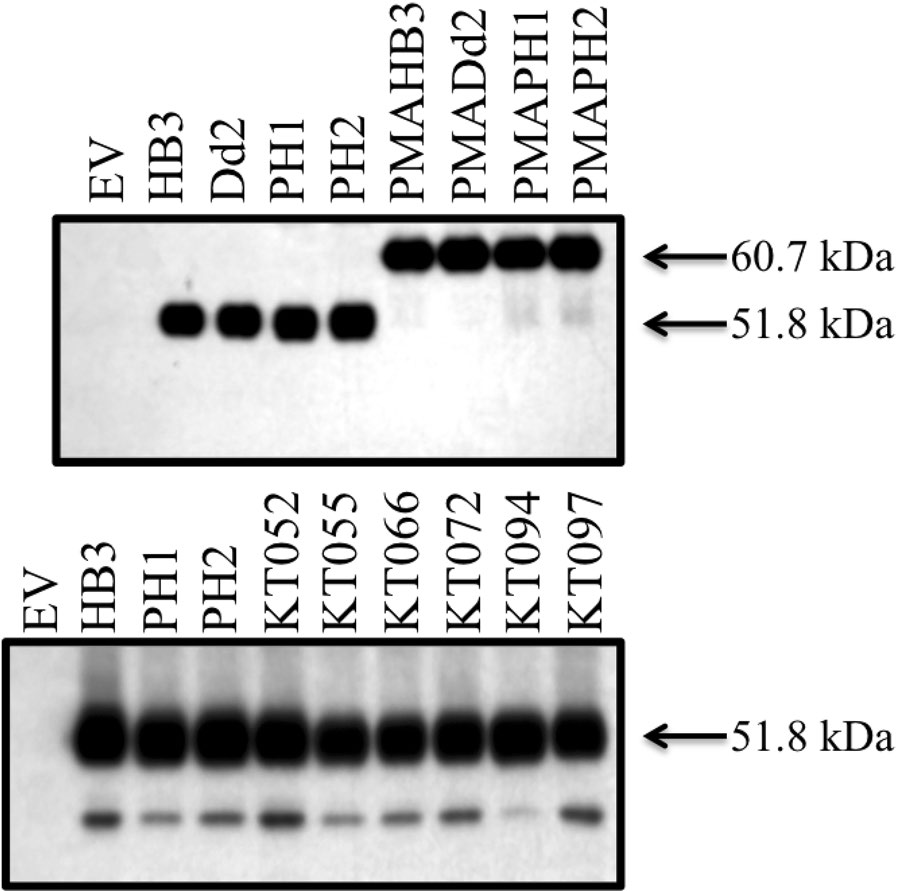
V5 Western blot quantifying relative PfCRT expression. *Blots* show approximately equal expression of indicated PfCRT and PMA-PfCRT isoforms in BY4741 (3 µg yeast membranes, *top*) and CH1305 (7 µg yeast membranes, *bottom*) yeast

As described in previous work, a rapid method for assaying CQ transport mediated by PfCRT protein expressed in growing yeast has been perfected [3, 4, 17]. In this method, PfCRT/CQ-dependent growth delay is linearly proportional to PfCRT-mediated CQ transport [3, 17]. Growth delay depends on the expression of PfCRT, the concentration of extracelluar CQ, and the magnitude of membrane potential across the yeast plasma membrane (PM) [3, 4, 17]. Expression of PfCRT alone, without including CQ in the yeast growth medium, conferred no change in growth rate and no other change in easily measureable yeast characteristics [3, 4, 17]. However, during the most recent screen of all known, naturally occurring PfCRT isoforms [4] the expression of two PfCRT isoforms, PH1 and PH2 (Table 1) [25–28], appeared to be intrinsically toxic to growing yeast, meaning expression of the proteins alone, without the presence of any antimalarial drug, conferred some degree of growth inhibition to yeast expressing these PfCRTs (e.g., Figure 1a compare lanes 3, 4 bottom panel to lanes 3, 4 top). Figure 1b further quantifies this growth delay in the absence of CQ for yeast expressing unmodified and modified (see below) PH1 and PH2 *vs* strains expressing HB3 and Dd2 PfCRT [3, 4, 17]. Altered levels of PH1 and PH2 PfCRT expression in these yeast, relative to other PfCRT isoforms, does not explain this intrinsic toxicity, since expression of these is approximately equal to that of all other PfCRT isoforms studied (Fig. 2; see also [4]). These results confounded initial attempts to quantify CQ transport catalyzed by PH1 and PH2 PfCRT, since the yeast-based methods rely on there being no effect of PfCRT expression alone [3, 4, 17].

**Table 2:** Oligonucleotides used in this study

	Sequence (5′–3′)
K76T	CAGTTTGCGTGATGAACACGATCTTCGCTAAGAGAAC
A144T	CTTGCAGCGTCATCTTGACCTTCATCGGTCTTACC
L160Y	CAGGTAACATTCAGTCCTTCGTCTATCAACTATCAATTCCAATCAACATG
N326D	CGCCTTGTTCTCATTCTTCGACATCTGTGATAACCTGAT
C72S	GTCCATCATCTACCTGTCAGTTTGCGTGATGAACAC
I166 V	TCGGTCTTACCAGAACCGCAGGTAACATTCAGTCC
H273 N	ATCTGTGATAACCTGATCAGCAGCTACATCATCGATAAG
L160P	CAGGTAACATTCAGTCCTTCGTCTATCAACTATCAATTCCAATCAACATG

**Fig. 3:**
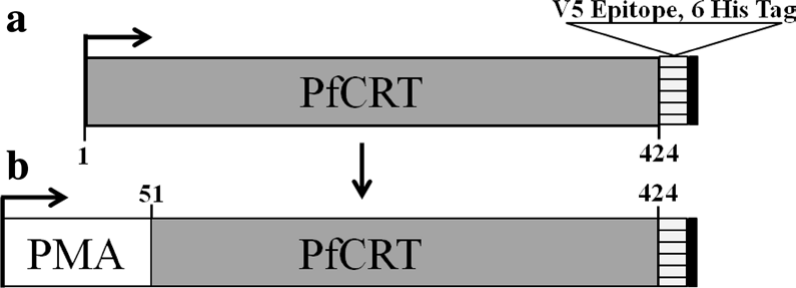
Diagram of PfCRT-vh and PMA-PfCRT-vh open reading frames. **a** Coding region for 424 amino acids of PfCRT (*grey*), 21 amino acid V5 epitope (*horizontal bars*) and 6His tag (*black*). **b** 25 N-terminal amino acids of PH1 and PH2 PfCRT were replaced with the 111 amino acid N-terminal leader sequence from yeast plasma membrane H^+^-ATPase (PMA, *white*)

**Fig. 4:**
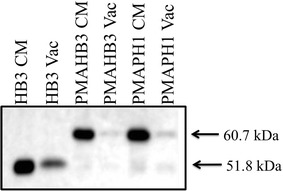
V5 western blot quantifying relative PfCRT expression in total yeast membranes (CM) vs isolated yeast vacuole membranes (Vac) for unmodified and and PMA-fused PfCRTs. All lanes contain 3 µg total protein. The *blot* shows that the PMA leader sequence substantially reduces PfCRT expression at the yeast vacuolar membrane relative to unmodified PfCRT (compare unmodified HB3 PfCRT, left two lanes, vs PMA-HB3 PfCRT chimera, middle two lanes). Note that we are unable to isolate purified vacuolar membranes for yeast expressing unmodified PH1 or PH2 PfCRT, since the intrinsic toxicity conferred by these PfCRTs leads to severe growth defects accompanied by serious perturbation of vacuolar function (and presumably vacuolar integrity) over longer periods of time in culture (see text)

PfCRT expressed in these yeast is localized primarily to the PM with low levels of expression at other endogenous internal membranes, principally the yeast vacuolar membrane [17]. To determine whether PH1 and PH2 PfCRT exert their toxicity via PM or vacuolar effects chimeras were created wherein PH1 and PH2 PfCRTs created using oligonucleotide (Table 2) site—directed mutagenesis were fused to the N-terminal leader sequence from the yeast plasma membrane H^+^-ATPase (PMA) (Fig. 3) as described previously [17]. Previous work demonstrated that such PMA-PfCRT fusion proteins remain functional and are localized more exclusively to the yeast plasma membrane [17], as is now also found for PH1 and PH2 chimeras (Fig. 4). Chimeras are expressed to similar levels, relative to the unmodified PfCRT isoforms (Fig. 2). Colony formation assays for yeast expressing these PH1- and PH2-PMA chimeras revealed significant mitigation of PH1 and PH2 PfCRT toxicity upon directed expression to the yeast PM (Fig. 1), indicating that low levels of vacuolarly localized PfCRT are the likely cause of the observed yeast toxicity.

**Fig. 5:**
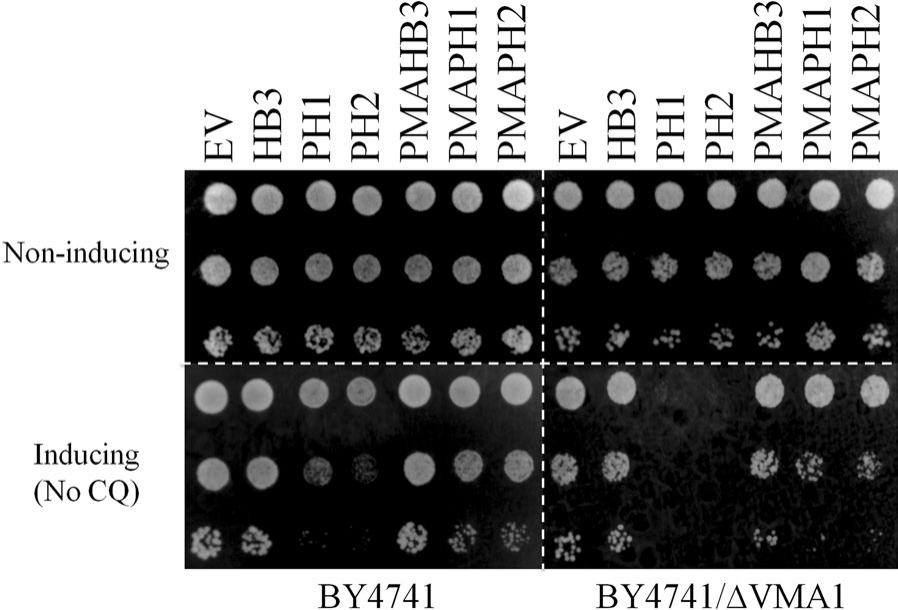
Colony formation assays for BY4741 and BY4741/Δvma yeast expressing the indicated PfCRT isoform, PMA-chimera, or empty vector (EV). *Top panels* show equal growth for all strains in non-inducing media. *Bottom left* (BY4741, inducing media) shows intrinsic toxicity of PH1 and PH2 expression in the BY4741 parental strain. *Bottom right* (BY4741/Δvma1, inducing media) shows conditional lethality of PH1 and PH2 expression in BY4741 yeast lacking vma subunit1

Perhaps not coincidentally, previous data showed that certain CQR-associated PfCRT isoforms perturb parasite digestive vacuolar (DV) physiology by decreasing DV pH and increasing DV volume [22, 23]. These effects are likely to due to perturbations in endogenous DV osmolyte traffic upon mutation of PfCRT [23, 29], suggesting that PH1 and PH2 PfCRT toxicity could be linked to perturbation of yeast vacuolar physiology caused by the small amounts of PfCRT expressed at the yeast vacuole membrane. To investigate this possibility, isoforms PH1 and PH2 were expressed in yeast lacking sub-unit 1 of the vacuolar membrane VMA (BY4741/Δvma1) since VMA function is essential for all other vacuolar physiology. Interestingly, PH1 and PH2 induction is lethal in a Δvma1 yeast background and lethality can be reversed by reducing expression within the yeast vacuolar membrane (Fig. 5, right panels; compare PH1 *vs* PMAPH1 and PH2 vs PMAPH2). The same lethal phenotype as well as the same mitigation of lethality is observed upon expression of these PH1/PH2 constructs in seven additional VMA sub-unit knockout yeast strains (see “Methods” and Table 3). These data suggest that low levels of PH1 and PH2 PfCRT vacuolar localization seriously impair residual VMA function for the weakened Δvma subunit strains.

**Table 3:** Vma subunit knockout strains used in this study

Systematic	Standard	Description
YDL185W	VMA1	Subunit A of the V1 peripheral membrane domain of the vacuolar H^+^-ATPase
YBR127C	VMA2	Subunit B of V1 peripheral membrane domain of the vacuolar H^+^-ATPase
YEL027W	VMA3	Proteolipid subunit C of the V0 domain of the vacuolar H^+^-ATPase
YOR332W	VMA4	Subunit E of the V1 peripheral membrane domain of the vacuolar H^+^-ATPase
YKL080W	VMA5	Subunit C of the V1 peripheral membrane domain of vacuolar H^+^-ATPase
YLR447C	VMA6	Subunit D of the V0 integral membrane domain of V-ATPase
YGR020C	VMA7	Subunit F of the V1 peripheral membrane domain of vacuolar H^+^-ATPase
YEL051W	VMA8	Subunit D of the V1 peripheral membrane domain of vacuolar H^+^-ATPase

Similar to results for the vma1 strain (see text) PH1 and PH2 expression was found to be synthetically lethal in each yeast strain lacking the designated subunit of yeast vacuolar (H^+^)-ATPase

**Fig. 6:**
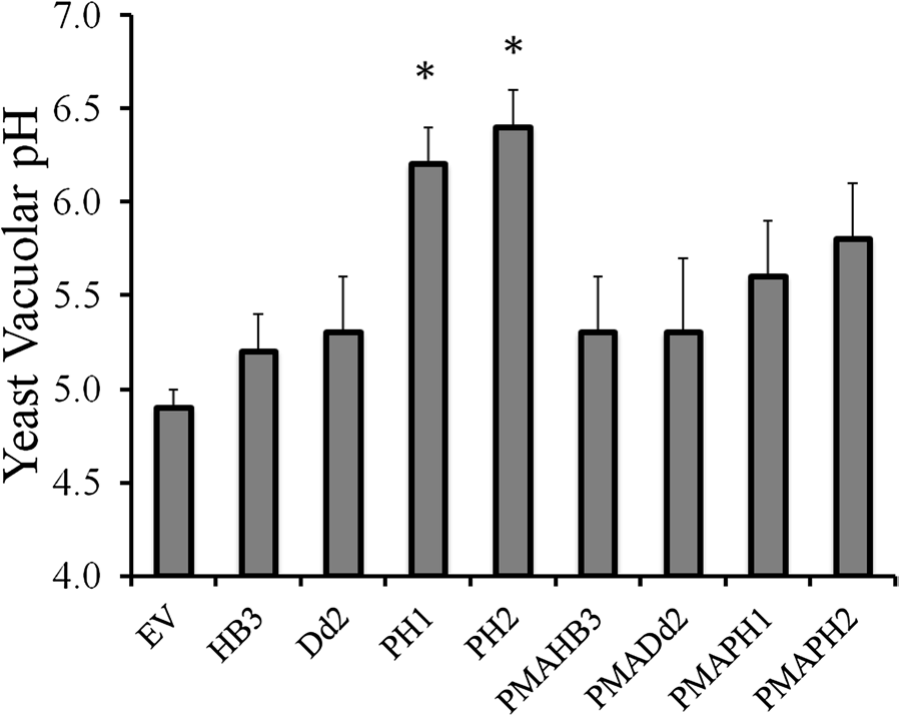
Yeast vacuolar pH. Vacuolar pH measured with BCECF-AM as described in “Methods” section for BY4741 yeast expressing indicated PfCRT isoform or empty vector. *Asterisk* indicates p value <0.05 vs empty vector

Yeast lacking VMA subunits exhibit a well-characterized ‘VMA phenotype’ resulting from impaired vacuolar acidification. This is typically defined by alkaline vacuolar pH and hypersensitivity to Ca^2+^ (measured as impaired growth in the presence of 50 mM CaCl_2_) [30, 31]. Alkaline vacuolar pH is an obvious consequence of impaired VMA function, and a properly acidified vacuole is required to energize the sequestration of high levels of free Ca^2+^. To determine if PH1 and PH2 expression results in an alkalinized yeast vacuole the probe BCECF-AM and previously published methods [18–20] were utilized to measure vacuolar pH for BY4741 yeast expressing PH1 and PH2 PfCRT (Fig. 6). PH1 and PH2 expression was found to result in more alkaline vacuolar pH relative to wild type yeast (transformed with an empty vector) as well as yeast expressing canonical CQS (isoform HB3) or CQR (isoform Dd2)—associated PfCRTs (Fig. 6). Once again, expression of PMA-fusion PH1 and PH2 attenuated the phenotype caused by expression of the unmodified PH1 and PH2 PfCRTs (Fig. 6).

**Fig. 7:**
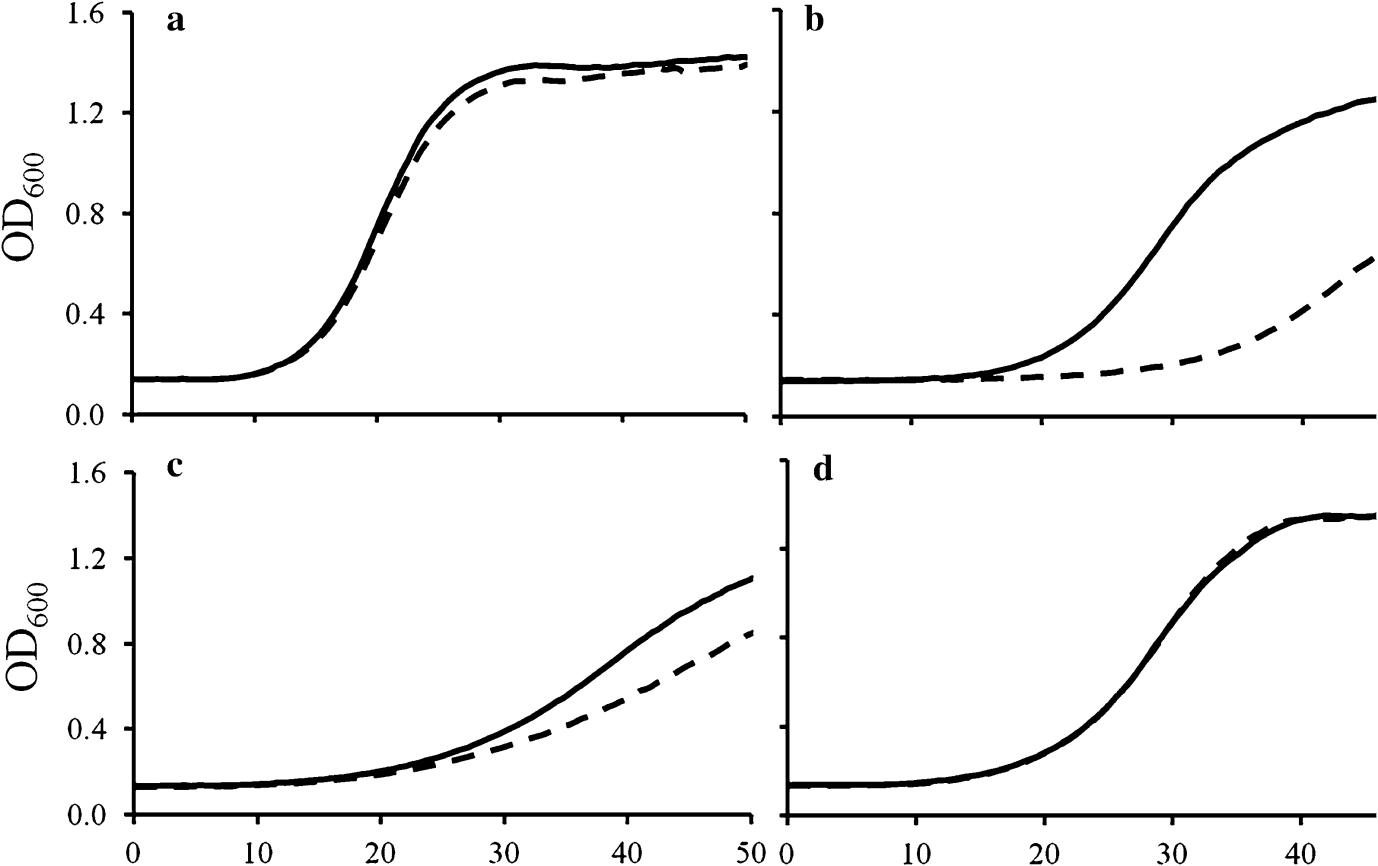
CaCl_2_ inhibits growth of Δvma1 and PH1-expressing yeast. BY4741/EV (**a**), BY4741Δvma1/EV (**b**), BY4741/PH1 (**c**), BY4741/PMAPH1 (**d**) growth in the presence of 0 mM (*black trace*) or 50 mM CaCl_2_ (*dashed trace*). Yeast strains were grown in media ± added 50 mM CaCl-_2_ in 96 well plates monitored by a Tecan automated plate reader as described [3, 4, 17]

When control BY4741 and BY4741/Δvma1 yeast are compared in the absence vs presence of 50 mM added Ca^2+^ (Fig. 7a vs b, black vs dashed lines respectively) Δvma Ca^2+^ hypersensitivity is readily apparent (compare dashed vs solid lines Fig. 7b vs a). Similarly, BY4741 yeast expressing PH1 PfCRT (Fig. 7c) or PH2 (not shown) showed increased Ca^2+^ sensitivity (compare dashed vs solid line, Fig. 7c). As expected, these sensitivities were again reversed by targeting PH1 and PH2 PfCRT localization to the PM (Fig. 7d). Note the intrinsic toxicity of PH1 is apparent by comparing black lines in Fig. 7c vs a, as is the mitigation of toxicity by comparing black lines in Fig. 7d vs c.

**Fig. 8:**
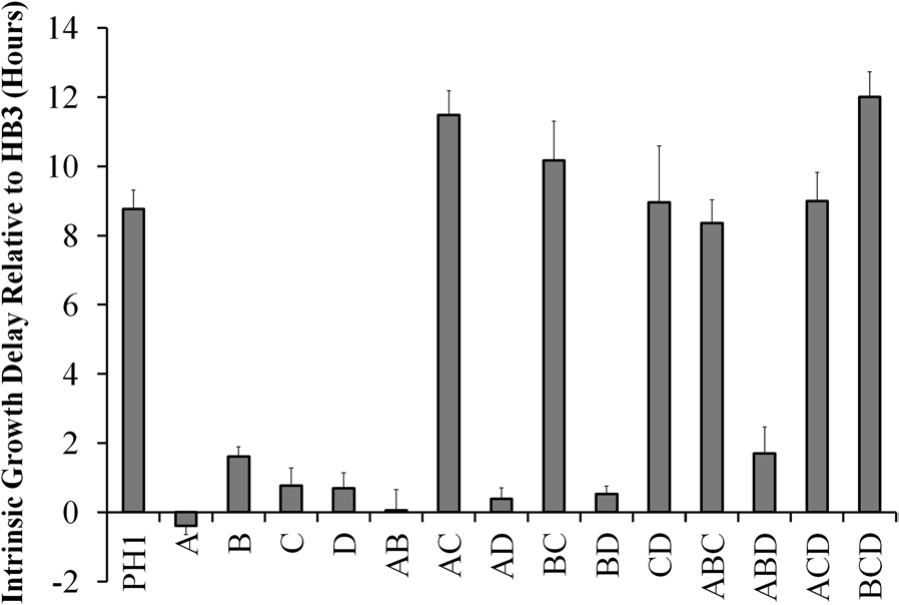
Intrinsic growth delays conferred by artificial (non natural) PfCRT isoforms with partial combinations of mutations that distinguish HB3 from PH1 PfCRT. Isoforms are named on the basis of mutations they harbour relative to HB3 PfCRT where A = K76T, B = A144T, C = L160Y, D = N326D. Growth delays reported relative to control yeast expressing HB3 PfCRT

PH1 PfCRT harbours only four amino acid substitutions relative to wild type (HB3 sequence, Table 1) and two of these (A144T and L160Y) are not found in other isoforms (Table 1) [4]. To determine the relative contribution of individual PH1 mutations to the intrinsic growth delay phenotype a systematic set of site-directed mutants representing every combination of the four amino acid substitutions that distinguish PH1 from HB3 (K76T, A144T, L160Y, N326D) were created. Interestingly, results indicate that the presence of the L160Y substitution, along with any one of the other amino acid substitutions, is required for PH1 toxicity (Fig. 8). Any substitution alone, or any other combination not involving L160Y, does not cause toxicity.

**Fig. 9:**
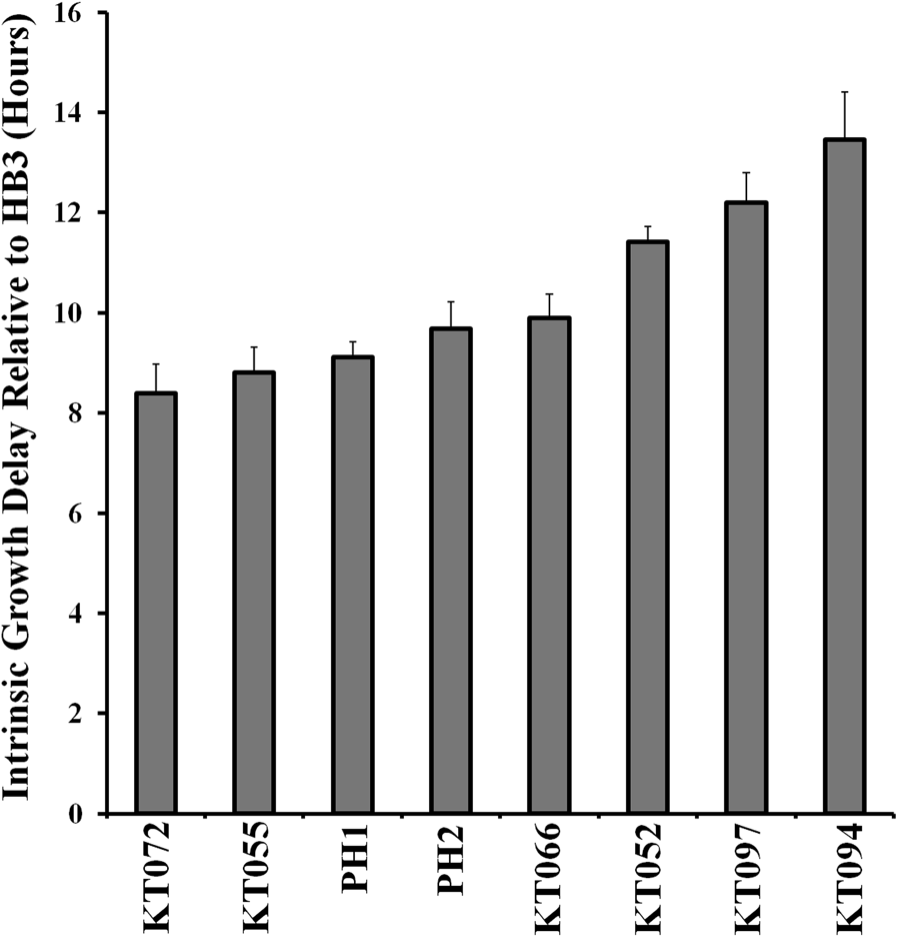
Intrinsic growth delay for yeast expressing the indicated PfCRT isoform

Interestingly, while this work was being conducted, a recent field study carried out in Sabah Malaysia identified 13 additional PfCRT isoforms [4], with six of these harbouring unique mutations A144T, L160Y, or L160P (Table 1) [28]. Due to the similarities to PH1 and PH2 isoforms these were screened and it was found that the six carrying unique mutations are also toxic to yeast, with isoform KT094 (L160P mutation; Table 1), conferring the highest toxicity yet measured (Fig. 9). The other 7/13 not carrying these mutations were not intrinsically toxic and are analysed and discussed elsewhere (see [4]). None of the 13 PfCRTs found in the isolates sequenced in this study [28] was found to harbour an exact PH1 or PH2 PfCRT sequence. Each of these new Malaysian isoforms either lacks one or more of the mutations contained in isoform PH2, or has acquired additional mutations (e.g., I166V and/or H273N; Table 1). Isoforms KT072 and KT055, which differ from PH2 with respect to a back-mutation towards wild type at position 326 (D326N) together with additional mutations I166V and H273N (KT072 only) conferred intrinsic growth delays that were slightly less than that conferred by PH2 (Fig. 9). It will prove interesting to monitor the relative prevalence of KT072/KT055 isoforms vs PH2 over time in this region of the globe.

**Fig. 10:**
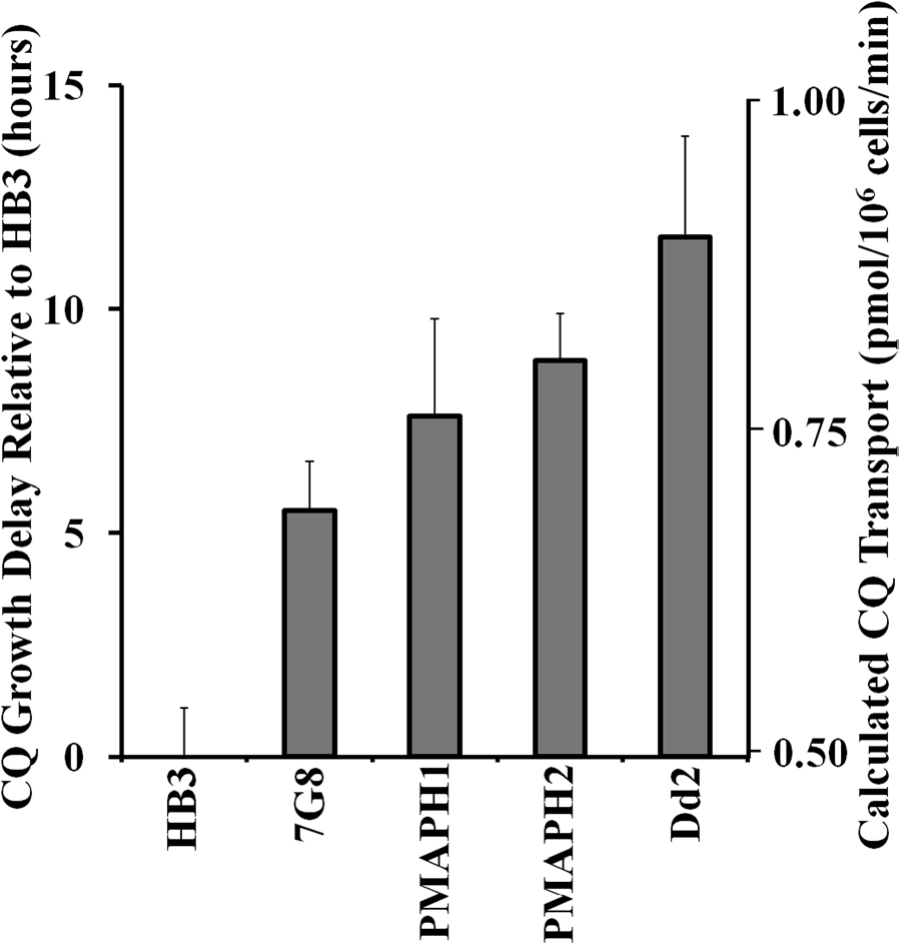
PfCRT induced growth delay in the presence of 16 mM CQ and calculated CQ transport catalyzed by PMA chimeras, vs previously measured transport for Dd2 and 7G8 PfCRT. Growth delays for PMA-PH1 and PMA-PH2 were measured as in Baro et al. [3] and converted to CQ transport using previously generated calibration curves as in Baro et al. [3]

The galactose-inducible PfCRT expression system can be used to measure PfCRT-mediated CQ transport, which has obvious direct significance with respect to parasite drug resistance. But, as mentioned above, the intrinsic toxicity of PH1 and PH2 in yeast would not allow unbiased measurement of a CQ transport phenotype. Therefore, growth delay for PMAPH1 and PMAPH2 chimera-expressing yeast was measured in the presence vs absence of CQ (Fig. 10) in order to calculate the transport of CQ mediated by these isoforms [3]. CQ transport was found to be intermediate to that catalyzed by CQR-conferring isoforms 7G8 and Dd2 (Fig. 10), predicting lower levels of CQR in PH1 and PH2—expressing *P. falciparum*.

To further test these conclusions parasite lines C8^PH1^ and C10^PH2^ were examined. These are gene edited derivatives of *P. falciparum* strain GC03 expressing PH1 and PH2 PfCRT recently created in the Fidock laboratory and have been reported on in more detail elsewhere [32]. Consistent with CQ transport mediated by PH1 and PH2 (Fig. 10) the gene-edited strains show intermediate levels of drug resistance ([32], D Fidock, pers. comm.). Because of the disruption to yeast vacuolar physiology caused by these isoforms potential changes to *P. falciparum* DV physiology for C8^PH1^ and C10^PH2^ were examined. Such changes might also help explain why these isoforms are relatively rare outside of the Philippines. Although the precise endogenous function of PfCRT remains unknown, hypotheses include ion, amino acid, glutathione and peptide transport from the DV [1, 29, 33–35]. Expression of CQS vs CQR isoforms of PfCRT have been shown to have different effects on DV pH and DV volume, as well as the accumulation of peptide fragments upon haemoglobin (Hb) proteolysis in some studies [22, 23, 29, 36]. Using previously published methods involving dextran-nerf exclusively entrapped within the DV [22, 23] DV pH and DV volume for PH1 and PH2 gene edited strains were measured to determine if these isoforms affected DV physiology in a manner distinct from control gene edited strains C2^GC03^ and C4^Dd2^, which harbour wild type and SEA CQR-conferring PfCRT isoforms, respectively. Interestingly, and although other transfectants expressing CQR PfCRT isoforms Dd2 and 7G8 were previously found to have more acidic DV pH [23], mild, but statistically significant alkalinization of DV pH for transfectant strains expressing PH1 and PH2 PfCRT (Table 4) was measured. Also in contrast to strains expressing other CQR-conferring PfCRTs [22], DV volume was found to be slightly smaller than DV volume for control transfectants expressing CQS-associated (HB3) PfCRT (Table 4).

**Table 4:** Digestive vacuole volume and pH of transfected parasite lines

Parasite line	PfCRT Isoform	DV Vol (µm^3^)	SEM	DV pH	SEM
GCO3^C2^	HB3	2.03	0.10	5.75	0.01
GCO3^C4^	Dd2	2.80	0.11	5.31	0.02
GCO3^C8^	PH1	1.61	0.04	5.84	0.04
GCO3^C10^	PH2	1.74	0.03	5.81	0.03

Values represent the average of three independent experiments per line

## Discussion

When taken together, data in this paper, along with other recent work [3, 4, 17], shows that PfCRT drug transport activity does not correlate with levels of CQR found in the cognate strain or isolate expressing that PfCRT isoform. This indicates that other factors, or other functions of PfCRT, complement the CQR phenotype conferred by PfCRT-mediated CQ transport. Previous data [4] also show that some PfCRT isoforms predicted to confer CQR via sequence analysis (e.g., the presence of K76T substitution) do not catalyze heightened CQ transport at all and are thus unlikely to confer CQR, consistent with other reports [10–12]. Data in this paper further suggest that certain combinations of mutations in K76T PfCRTs (particularly those involving residues 144 and 160) very significantly effect CQ transport.

In all previous experiments analysing PfCRT isoform function in yeast, neither the presence of external CQ alone or PfCRT expression alone conferred any significant change in yeast growth rates, whereas the presence of both PfCRT and external CQ leading to increased inward transport of toxic CQ dramatically slowed yeast growth [3, 17]. In contrast, eight isoforms of PfCRT that harbour an unusual mutation at residue 160 (two from Philippines, six from Malaysia) are now found to be intrinsically toxic to yeast, since they slow growth when expressed even in the absence of CQ. The intrinsic yeast toxicity of these unusual mutant isoforms is the result of an alteration or dysregulation of the yet incompletely described endogenous function of PfCRT. Thus, CQ transport could not be easily quantified for the interesting PH1 or PH2 PfCRT isoforms in their unmodified form. Nonetheless, transport for PMAPH1 and PMAPH2 chimeras was quantified (Fig. 10) and showed that these isoforms transport CQ with an efficiency that is intermediate to transport found previously for CQR isoforms 7G8 and Dd2. This is consistent with previous CQ transport results for these same isoforms expressed in oocytes [37] and with initial measurement of the levels of CQR conferred by these isoforms in *P. falciparum* transfectants [32].

Further experiments indicated that targeted expression of PH1 and PH2 PfCRT to the yeast PM substantially reduced the toxicity of these isoforms (Figs. 1, 4). This suggested that the bulk of PH1 and PH2 PfCRT toxicity is levied from a different localization. Given that PfCRT is being over-expressed in this yeast model system and that the vacuolar membrane harbours the second most abundant amount of PfCRT [17], it was suspected that PH1 and PH2 PfCRT within the vacuolar membrane caused the intrinsically toxic phenotype. The lethality of PH1 and PH2 expression in Δvma yeast strains provided additional evidence for this hypothesis, as did observed elevated yeast vacuolar pH and increased yeast sensitivity to Ca^2+^ (hallmarks of a dysregulated yeast vacuole), all of which were then reversed for the PMAPH1 or PMAPH2 chimeras. Analysis of a systematic set of artificial mutants harbouring every possible combination of PH1 mutations indeed indicated that it is mutation at L160 (observed only in these eight out of 53 total known PfCRT isoforms) that is obligate for conferring the intrinsic toxicity. Interestingly however, mutation L160Y has a very small effect on PfCRT function when present alone and only creates an intrinsically toxic PfCRT when paired with any one of the other three PH1 mutations (Fig. 9). Analysis of isoform KT094 provided additional evidence pointing to the unique importance of residue 160. KT094 harbours a L160P mutation and a wild type 144A yet confers the largest growth delay of any of the natural isoforms yet examined.

Field studies measuring the prevalence of wild type *vs* CQR-conferring PfCRT isoforms have found that in some areas where CQ pressure has been removed, the percentage of parasites harbouring wild type PfCRT has increased relative to parasites harbouring Old World CQR-associated isoforms (e.g., Dd2 and FCB isoforms of SEA origin), but not relative to New World CQR isoforms (e.g., 7G8 and PNG4 from SA and PNG) [8, 38–40]. This phenomenon has been cited as evidence for a fitness cost associated with isoforms that have amino acid residue 72–76 sequence = CVIET, but not = SVMNT (Table 1) [4]. The spread of SVMNT isoforms over the past 20 years provides additional evidence in support of this hypothesis. PH1 and PH2 isoforms represent a unique case in that they indeed more closely resemble New World isoforms, and are common within the Philippines, but nonetheless have not yet spread geographically beyond the Philippines. In fact, several field studies in Cambodia, Vietnam and China have been devoted to the search for mutations A144T and L160Y and while these studies have led to the discovery of several new isoforms with novel mutations at residue 144 (A144F, A144Y), no isoforms harbouring mutations at residue 160 have been found [10, 41, 42]. In contrast, a very recent field study from Sabah, Malaysia, which shares a maritime border with the Philippines, reported the isolation of six parasites expressing PfCRT isoforms with mutations to residue 160 (Table 1) [28]. However, none of these isoforms is an exact match to PH1 or PH2 PfCRT. Two of these isoforms (KT055 and KT072) conferred less yeast toxicity relative to PH2 PfCRT from which they are probably derived. This may provide additional evidence that PH1 and PH2 PfCRT-bearing parasites are less stable or cannot thrive outside Filipino drug use practices without the acquisition of compensatory mutations (e.g., I166V and H273N). It is worth noting that these putative compensatory mutations are also present in PfCRT orthologues from other malaria species for which no link between PfCRT mutation and CQR has yet been established. Mutation I166V is found in wild type *P. chabaudi* and *P. yoelii* PfCRTs and mutation H273 N is found for PfCRT expressed in *P. vivax* and *P. knowlesi.* It is possible that these residues are required to restore or better maintain endogenous physiological PfCRT function in the presence of other CQR-conferring mutations.

Consistent with perturbations in yeast vacuolar physiology, results from transfected *P. falciparum* lines harbouring PH1 and PH2 PfCRT reveal that expression of either isoform also alters pH and reduces DV volume for mid stage trophozoites (Table 4). Previous studies have shown that *P. falciparum*-transfected lines expressing Dd2 or 7G8 PfCRT have increased DV volume [22] and analysis of PfCRT homologue TgCRT from *Toxoplasma gondii* demonstrated that reduced TgCRT expression yielded an increase in the volume of that parasite’s vacuole (an internal acidic organelle similar to the DV) [43]. It has been suggested that the increased volume phenotype in these examples is indicative of impaired or reduced normal physiological function of the PfCRT resulting from either eight amino acid substitutions required for CQR (Dd2) or decreased levels of TgCRT. In theory, reduced transport of PfCRT’s physiological substrate (possibly Hb-derived peptides [29]) would result in substrate accumulation within the DV and a concomitant increase in DV volume. By the same reasoning then, perhaps upregulated endogenous transport function by PH1 and PH2 PfCRT is reducing internal PfCRT substrate concentration relative to wild type such that DV volume would then decrease. Consistent with this hypothesis, bioinformatic analysis of PfCRT homologues places mutations A144T and L160Y at sites predicted to be important for translocation and binding of substrate, respectively [44]. Further studies are required to test this idea by determining if PH1 and PH2 isoforms alter the peptide accumulation phenotype associated with Dd2 PfCRT [29]. However, additional data showing yeast alkaline vacuolar pH due to PH1 and PH2 predicts that Hb-derived peptides are unlikely to be the only substrate for PfCRT, since these do not exist in the yeast vacuole. The results of Lewis et al. [29] actually do not require that PfCRT transport peptides at all. Alternatively, since the proteases that cleave Hb to create these peptides are highly pH dependent, altered DV pH caused by mutation of PfCRT could also produce the results seen by Lewis et al. [29]. This reasoning is attractive since it does not envision that PfCRT recognizes a wide variety of structurally divergent peptides, dipeptides, or amino acids. Presumably alterations in yeast vacuole pH caused by PH1 and PH2 PfCRT are due to altered ion transport as also proposed for other mutant PfCRTs [22, 35, 45, 46], perhaps heightened outward movement of H^+^ relative to HB3 and Dd2 PfCRT.

## Conclusions

Recently, CQ transport for 45 of the 53 naturally occurring isoforms of PfCRT has been measured and found to have a wide range of activities that predict a range of CQ sensitivities for *P. falciparum* isolates expressing these isoforms [3, 4, 17]. In this work the remaining eight known, naturally occurring, isoforms of PfCRT (all of which harbour a substitution at residue 160) were examined. Along with data in this paper, there is also evidence from other studies that PfCRT residues at or near position 160 are critical for proper balance between parasite fitness characteristics and drug resistance [48–50]. The eight isoforms studied here are unusual not only in their pattern of amino acid substitutions, but also because they show varying levels of intrinsic toxicity when expressed in yeast, most likely through significant disruption of yeast vacuolar physiology. This behaviour is distinct from that of the other 45 naturally occurring PfCRT isoforms [3, 4], yet further highlights the role that PfCRT plays in maintaining critical vacuolar properties [22, 29, 48]. Previously, acidic DV pH and larger DV volume have been found for some CQR strains expressing certain CQR—associated mutant PfCRT isoforms [23, 24], but additional results in this paper suggest that other CQR—associated mutant PfCRTs can exert reciprocal effects on these parameters. As previously suggested these physiologic perturbations of DV pH and volume likely add together with CQ transport catalyzed by PfCRT to further affect levels of CQR [33]. Lower DV pH can indeed promote CQR by leading to aggregation of the CQ target (free DV haem) as previously suggested [47] and elevated DV pH would be predicted to add to CQR by lowering DV concentrations of CQ via the predictions of weak base partitioning [51]. More work defining the magnitude of these physiological changes in other transfectants expressing other PfCRT isoforms, and in calibrating the precise contributions that they make to CQR, is needed to further test these theories.

## Abbreviations

CQ: chloroquine
CQR: chloroquine resistant
CQS: chloroquine sensitive
CS: cytostatic
DV: digestive vacuole
Hb: haemoglobin
PfCRT: *Plasmodium falciparum* chloroquine resistance transporter
PM: plasma membrane
PMA: plasma membrane H+-ATPase
PNG: Papua New Guinea
SA: South America
SCP: single-cell photometry
SEA: Southeast Asia
VMA: yeast vacuolar membrane H+-ATPase
